# Responsiveness of pain, functional capacity tests, and disability level in individuals with chronic nonspecific low back pain

**DOI:** 10.1142/S101370252050002X

**Published:** 2019-12-06

**Authors:** Prasert Sakulsriprasert, Roongtiwa Vachalathiti, Pathaimas Kingcha

**Affiliations:** 1 Division of Physical Therapy, Faculty of Physical Therapy, Mahidol University, Nakhon Pathom, 73170, Thailand; 2 Physical Therapy Center, Faculty of Physical Therapy, Mahidol University, Nakhon Pathom, 73170, Thailand; roongtiwa.vac@mahidol.ac.th

**Keywords:** Back pain, functional, test, capacity, sensitivity, disability

## Abstract

**Background:**

Clinical outcomes are very important in clinical assessment, and responsiveness is a component inside the outcome measures that needs to be investigated, particularly in chronic nonspecific low back pain (CNSLBP).

**Objective:**

This study aimed to investigate the responsiveness of pain, functional capacity tests, and disability in individuals with CNSLBP.

**Methods:**

Twenty subjects were assessed in pain using the following methods: visual analog scale (VAS) and numeric pain rating scale (NPRS), functional capacity tests: functional reach test (FRT), five-time sit-to-stand test (5 TSST), and two-minute step test (2 MST), and disability level: modified Oswestry Disability Questionnaire (MODQ), Thai version before and after 2-week intervention session. For interventions, the subjects received education, spinal manipulative therapy, and individual therapeutic exercise twice a week, for a total of two weeks. The statistics analyzed were change scores, effect size (ES), and standardized response mean (SRM).

**Results:**

The most responsive parameter for individuals with CNSLBP was pain as measured by numeric pain rating scale (NPRS) (ES −0.986, SRM −0.928) and five-time sit-to-stand test (5 TSST) (SRM −0.846).

**Conclusion:**

This study found that NPRS pain and 5 TSST were responsive in individuals with CNSLBP at two weeks after the beginning of interventions.

## Introduction

Low back pain (LBP) is a major health problem internationally characterized by a range of biophysical, psychological, and social dimensions affecting functioning, societal participation, and personal financial prosperity.^1,2^ The reported prevalence of LBP was high, especially for chronic nonspecific low back pain (CNSLBP), approximately 15.4% (Ref. 3) of which chronic low back pain was about 2.5 times more prevalent in working population compared to nonworking group.^4^ Nonspecific low back pain is labeled so when the specific nociceptive source cannot be found^5^ while chronic is defined so when the duration that the pain persists is longer than 3 months.^6^ As aforementioned impacts are caused by LBP, the outcome assessments for individuals with LBP should therefore cover pain assessment, and also related activities and disability.

Responsiveness is very important for clinicians to consider when the outcome measures are used clinically, by which the responsiveness is the ability of the outcome measures to detect the patient’s recovery or health status over time.^7^ The recommended methods for statistical analysis to represent the responsiveness consist of change score, effect size (ES), and standardized response mean (SRM).^8-11^ It has been reported that for the individuals with acute nonspecific low back pain, the responsive outcomes were pain and disability as reported with ES. In addition, it was found that most of the patients recovered in 2 weeks,^12^ to be comparable to the patients with acute LBP, the duration of 2 weeks between the baseline and after-intervention assessments gains attention for the study in the patients with chronic LBP. For patients with chronic LBP, the responsiveness has been studied in various outcome measures such as SF-36 and cooperative (COOP) chart system,^13^ Oswestry Disability Index (ODI), EuroQol (EQ-5D), and Shuttle Walking Test (SWT).^14^ However, the previous studies had the heterogeneity of the recruited patients, different time interval examinations, and various statistical analyses.^13,14^ In addition, the test such as SWT has some limitations for clinical use because of the acceptability to patients and the cost for administration.^14^ However, the functional tests are very important for the assessment because they can represent the individual’s capacity for performing particular activities. The concepts of functional capacity tests have been developed by Simmonds *et al.*, by using the standardized pattern of functional tests, while minimal equipment is needed and the administration and interpretation are simple. The exemplary functional capacity tests are functional reach test (FRT), 5 TSST, and 2 MST.^15^ These functional capacity tests have advantages for clinical use, but the responsiveness of each test needs to be investigated.

This study was therefore conducted to examine the responsiveness of pain, functional capacity test, and disability level in individuals with CNSLBP on 2-week interval of pre-test and post-test assessments for determining the responsiveness of the selected clinical outcome measures.

## Materials and Methods

### Subjects

Twenty individuals with CNSLBP with the duration of their symptoms being at least 3 months with mild to moderate pain intensity (1–6 cm on visual analog scale, VAS) were recruited from Physical Therapy Center, Faculty of Physical Therapy, Mahidol University. The exclusion criteria were specific for radicular LBP, neurological or cardiovascular diseases, history of previous surgery at the spine or lower extremity, pregnancy, and on menstruation. Written informed consent was obtained from each individual before participation. The study protocol and informed consent have been approved by Mahidol University-Central Institutional Review Board (MU-CIRB), COA no. 2017/155.2808.

### Outcome measures

**Pain intensity:** Visual analog scale (VAS) with 10 cm horizontal line anchored by ‘no pain’ on the left end and ‘worst pain imaginable’ on the right was used. NPRS from 0 to 10 representing pain intensity verbally was also used in this study.^16,17^ The individuals with CNSLBP reported their pain intensity on worst movement or activity on the tested day by marking on VAS and verbal expression for NPRS.

### Functional capacity tests

There were three functional capacity tests in this study comprising functional reach test, five-time sit-to-stand test, and two-minute step test. The individuals with CNSLBP in comfortable clothes and canvas shoes were given an explaination and saw the demonstration before the test.

FRT was used for assessing dynamic balance and flexibility of the trunk muscle. The researcher attached a ruler to the wall at participant’s shoulder height and provided feet placement marks on the floor. The individuals with CNSLBP stood sideways next to the wall without leaning against the wall, feet apart as shoulder width, and raised both hands to 90^∘^, kept elbows straight and hands fisting and the 3rd metacarpal head on the ruler was recorded as the starting position. They were then instructed to reach forward with arm outstretched remaining in shoulder height, as far as possible without stepping three times, the best distance reached was then recorded.^18^

5 TSST was used for assessing back and lower limb strength. The researcher placed a chair against the wall for fixing the tested location. The individuals with CNSLBP were seated in the middle of the chair, back straight without support on the backrest and feet flat on the ground, both arms crossed to the chest. They were instructed to rise to fully stand and then returned to a fully seated position as fast as possible five times. The time spent to complete five times was recorded.^19^

2 MST was used to measure the endurance during dynamic weight shifting activity. The researcher measured the stepping height of each individual which was equal to the mid-thigh level, halfway between the iliac crest and patella, and marked the level on the wall. The individual was then informed to step in the provided place with moving the knee up to the predetermined mark on the wall alternately, started with the right leg and continued as many steps as possible within 2 minutes. The researcher counted the number of times the right knee reached the mark. If needed, the individual could be allowed to place one hand on the table for balance.^19^

**Disability level:** The total score of modified Oswestry Disability Questionnaire (MODQ), Thai version, has been used by summarizing from 10 items; pain intensity, personal care, lifting, walking, sitting, standing, sleeping, social life, traveling, employment/home making, which is categorized into 6 levels of each item starting from 0 (no disability) to 5 (highest disability) and multiplied with two to gain the percentage of disability level.^20^ It can imply how pain affects various activities of daily living.^20-22^ The higher percentage represented a greater level of disability defined as follows: 0–20 minimal disability, 21–40 moderate disability, 41–60 severe disability, 61–80 crippled, and 81–100 bed bound or symptom magnifier.^21^

### Procedures

The individuals were assessed two times, the first time was at the beginning of the intervention program, baseline or pre-test assessment. The second was at the end of the 2-week program, post-test assessment. The assessments were pain intensity during worst movement by VAS and NPRS, functional capacity tests comprising FRT 5 TSST, and 2 MST, and disability level by MODQ, Thai version. All assessments had been done by a physical therapist who has been trained for all assessments for 1 week of didactic period and training session before this study. After finishing pre-test assessment, the results were taken away and kept by another researcher. The physical therapy intervention program was conducted in interim between the two-time assessments. The intervention program comprising education, spinal manipulative therapy and therapeutic exercise^23^ including stretching and strengthening for lower back twice a week lasted for two weeks.

### Statistical analysis

The descriptive statistics for demographic data of the subjects were shown in number, means and standard deviations, and percentage. The responsiveness in this study consisted of change score, effect size, and standardized response mean. Change scores were calculated by subtracting post-test value from pre-test value, representing the magnitude of the difference between pre-test and post-test, the greater the magnitude, the greater the responsiveness or change. In this study, the change score was calculated as post-test data minus baseline data. ES has been recommended for determining the responsiveness.^24^ The calculation is the change score divided by standard deviation of the baseline score,^25^ the value below 0.2 is considered small, 0.5 moderate, and 0.8 large according to previous studies.^8,10,11^ SRM is similar to ES, which is calculated by the change score divided by standard deviation of the change. Therefore, SRM indicates an estimate of change, which is standardized relative to the variability in change scores. The consideration for the calculated values is the same as ES, or 0.2, 0.5, and 0.8 representing small, moderate, and large, respectively.^9^ To compare the change scores between VAS and NPRS, the independent t-test was used since the data were normally distributed with SPSS program version 23 (IBM Corp., Armonk, NY), the statistical significance was set at p-value less than 0.05.

## Results

Demographic data of all subjects are reported in Table 1. Baseline and post-test data of the subjects are shown in Table 2. The results of change score, ES, and SRM for all parameters are provided in Table 3. The change scores of pain intensity were −1.3 cm and −1.6 cm in VAS and NPRS, respectively. The change scores of functional capacity tests were 0.9 cm for FRT, −1.5 s for 5TSST, and 14.5 repetitions for 2MST, respectively. The change score of disability as measured by MODQ total score was −5.1%.

**Table 1.** Demographic data of the subjects.

Characteristics	Summary
Female/Male, number	13/7
Age, years	43.15 ± 2.03
Weight, kg	57.80 ± 2.06
Height, cm	161.40 ± 1.69
BMI, kg/m^2^	22.18 ± 0.60
Duration of symptom, months	32.30 ± 35.50
**Location of LBP, %**	
Left or RightLeft side	35%
Right side	20%
Both sides or Centralized	45%
Sacroiliac joint involvement	
Yes	15%
No	85%
**Working tasks, %**	
Doing housework	10%
Working in prolonged sitting	65%
Working in prolonged standing	25%

**Table 2.** Baseline and post-test data of the subjects.

Parameters	Baseline	Post-test
**Pain**		
Visual analog scale (VAS), cm	3.2 ± 1.6	1.9 ± 1.9
Numeric pain rating scale (NPRS), (0–10)	4.0 ± 1.6	2.4 ± 1.9
**Functional capacity test**		
Functional reach test (FRT), cm	34.4 ± 5.7	35.3 ± 6.1
Five-time sit to stand test (5 TSST), sec	11.3 ± 3.0	9.8 ± 3.2
Two-minute step test (2 MST), rep	77.4 ± 34.0	91.9 ± 23.7
**Disability**		
MODQ total score, %	14.0 ± 11.3	8.9 ± 7.9

**Table 3.** Change score, effect size, and standardized response mean of each parameter.

Parameters	Change score	Effect size	Standardized response mean
Visual analog scale (VAS)	−1.3	−0.789	−0.781
Numeric pain rating scale (NPRS)	−1.6	−0.986	−0.928
Functional reach test (FRT)	0.9	0.158	0.184
Five-time sit to stand test (5 TSST)	−1.5	−0.510	−0.846
Two-minute step test (2 MST)	14.5	0.424	0.583
MODQ total score	−5.1	−0.448	−0.487

For the ES, the most responsive parameters were pain measured by NPRS (ES=−0.986), and VAS (ES=−0.789). While 5TSST had the highest responsiveness for functional capacity test (ES=−0.510).

For the SRM, the most responsive parameter was NPRS (SRM=−0.928), followed by 5TSST (SRM=−0.846), and VAS (SRM=−0.781).

The comparison of the change scores between VAS and NPRS showed no significant difference, t=0.559, p-value was 0.580.

## Discussion

This study aimed to investigate the responsiveness of clinical outcomes regarding pain, functional capacity tests, and disability level in individuals with CNSLBP over the period of 2weeks. The results of this study showed that pain as reported by VAS and NPRS were responsive according to their change scores, ES, and SRM. Using Cohen’s suggestions,^8^ the value of 0.8 or more is considered large for ES and SRM. The ES and SRM of NPRS were −0.986 and −0.928, respectively. These values were therefore construed as large and responsive. While VAS was little lower than NPRS in terms of change score, ES, and SRM. However, the comparison of the change scores between VAS and NPRS showed no significant difference. In addition, it has been proved that VAS and NPRS have good agreement when using both in acute pain assessment.^26^ The pain assessment in patients with chronic LBP contains multiple factors. This study therefore used the functional capacity tests and the disability level additionally for better understanding of the changes in the patients with CNSLBP.

The functional capacity tests, according to SRM, 5TSST were proved to be most responsive rather than FRT and two-minute step test. This result might be because 5TSST involves the strength of knee extensor in performing standing up together with the activity of back extensor muscles for adjusting the trunk upright by extensor moment.^27^ This responsiveness could be hypothesized such that the function of back extensor muscles was perhaps better at the post-test examination on the period of 2 weeks owing to decreased pain contributing to increased speed of movement, however, the change of back extensor strength should be investigated for further study.

While FRT and 2MST had small to moderate responsiveness reported as ES and SRM. FRT involves the dynamic postural control for shifting the center of mass (COM) towards the front edge of the base of support as needed.^18,28^ Also, 2MST was used to investigate the dynamic postural control together with aerobic endurance requiring weight shifting, lower limb movement, and stability of the spine.^19^ Their lower responsiveness results could indicate that the improvement of dynamic postural control needs longer period than 2 weeks for this training in individuals with CNSLBP, the results therefore suggested the practitioners or therapists to understand the recovery and set proper therapeutic time frame since the responsiveness in dynamic postural control would take longer time to follow-up as measured by FRT and 2 MST. In addition, the intervention program in this study did not include the dynamic postural control training, the active portion of the program was back stretching and strengthening exercise. The small responsiveness data might represent the exercise specificity while the back strengthening exercise had minimal cross-over effect to dynamic postural control. The intervention program for future study might include also the dynamic postural control training if the assessment pertaining to the dynamic postural control is included.

For disability level, the responsiveness data using MODQ total score as reported with change score, ES, and SRM were −5.1, −0.448, and −0.487, respectively, which were smaller than the previous study^12^ with change score −16.2 and ES −0.930. The result could clearly be concluded as that the individuals with CNSLBP had slower recovery as represented by decreased disability level. Therefore, the future study should provide longer duration of post-test assessment for better result of responsiveness in disability level. This study was designed to be 2-week interval assessment in order to be comparable to the previous study,^12^ by which using the same methods of statistical analysis for responsiveness, also, the clinical setting and intervention program were also as same as the previous study to investigate the behavior of the change in individuals with CNSLBP compared to the acute patients that had been taken as reference. The results in this study were therefore can be concluded such that the responsiveness in individuals with CNSLBP was lower than patients with acute LBP in the clinical outcomes such as pain and disability level.

One of the limitations in this study is the responsiveness analyses, which was internal responsiveness. The external responsiveness was not studied since another relevant outcome measure was needed for correlation. However, the individuals with CNSLBP had many clinical aspects which were difficult to determine, requiring another relevant outcome to cover all. Another consideration was the time interval. The future study should take longer period for the responsiveness analysis to see greater change representing larger responsiveness.

## Conclusion

This study investigated the responsiveness of the clinical outcomes used to measure the changes in individuals with CNSLBP at 2 weeks after the beginning of interventions.

The most responsive parameter in this study was pain as presented with the highest values in ES and standardized response mean. While the functional capacity tests were less responsive than pain, the longer duration of physical therapy intervention aiming to promote functional capacity is needed which is also suggested for further investigation.

## Acknowledgments

The authors would like to thank all participants in this study from Physical Therapy Center, Faculty of Physical Therapy, Mahidol University, Thailand.

## Conflict of Interest

The authors declare no conflict of interest.

## Author Contributions

The preparation of the paper along with the literature review and data analysis was carried out by Prasert Sakulsriprasert.

Roongtiwa Vachalathiti helped in preparation of the paper, research design planning, results validation and research team training. Pathaimas Kingcha helped in data collection, literature review and team coordination.

## References

[1] Hartvigsen J, Hancock M, Kongsted A, Louw Q, Ferreira M, Genevay S, What low back pain is and why we need to pay attention. The Lancet 2018;391(10137):2356–67. 10.1016/S0140-6736(18)30480-X 29573870

[2] Koes B, van Tulder M, Ostelo R. Clinical guidelines for the management of low back pain in primary care. Spine 2001;26(22):2504–14. 11707719 10.1097/00007632-200111150-00022

[3] Iizuka Y, Iizuka H, Mieda T, Tsunoda D, Sasaki T, Tajika T, Prevalence of chronic nonspecific low back pain and its associated factors among middle-aged and elderly people: An analysis based on data from a musculoskeletal examination in Japan. Asian Spine J 2017;11(6):989–97. 29279756 10.4184/asj.2017.11.6.989 PMC5738322

[4] Jackson T, Thomas S, Stabile V, Shotwell M, Han X, McQueen K, A systematic review and meta-analysis of the global burden of chronic pain without clear etiology in low- and middle-income countries: Trends in heterogeneous data and a proposal for new assessment methods. Anesth Analg 2016;123:739–48. 27537761 10.1213/ANE.0000000000001389

[5] Maher C, Underwood M, Buchbinder R Non-specific low back pain. Lancet 2017;389:736–47. 27745712 10.1016/S0140-6736(16)30970-9

[6] Chou R, Shekelle P Will this patient develop persistent disabling low back pain? JAMA 2010;303:1295–302. 20371789 10.1001/jama.2010.344

[7] Portney L, Watkins M Foundations of clinical research: Applications to practice. 2nd ed. Upper Saddle River, New Jersey: Prentice-Hall, Inc., 2000.

[8] Cohen J Statistical Power Analysis for the Behavioural Sciences. New York: Academic Press, 1977.

[9] Liang M, Fossel A, Larson M Comparisons of five health status instruments for orthopedic evaluation. Med Care. 1990;28:632–42. 2366602 10.1097/00005650-199007000-00008

[10] Norman G, Stratford P, Regehr G Methodological problems in the retrospective computation of responsiveness to change: The lesson of Cronbach. J Clin Epidemiol. 1997;50:869–79. 9291871 10.1016/s0895-4356(97)00097-8

[11] Stucki G, Liang M, Fossel A, Katz J Relative responsiveness of condition-specific and generic health status measures in degenerative lumbar spinal stenosis. J Clin Epidemiol 1995;48:1369–78. 7490600 10.1016/0895-4356(95)00054-2

[12] Sakulsriprasert P, Vachalathiti R, Vongsirinavarat M, Pichaisak W Responsiveness of pain, active range of motion, and disability in patients with acute nonspecific low back pain. Hong Kong Physiother J. 2011;29:20–4.

[13] Bronfort G, Bouter L Responsiveness of general health status in chronic low back pain: A comparison of the COOP Charts and the SF-36. Pain. 1999;83:201–9. 10534591 10.1016/s0304-3959(99)00103-7

[14] Campbell H, Rivero-Arias O, Johnston K, Gray A, Fairbank J, Frost H Responsiveness of objective, disease-specific, and generic outcome measures in patients with chronic low back pain: An assessment for improving, stable, and deteriorating patients. Spine. 2006;31(7):815–22. 16582856 10.1097/01.brs.0000207257.64215.03

[15] Simmonds M, Olson S, Jones S, Hussein T, Lee C, Novy D, Psychometric characteristics and clinical usefulness of physical performance tests in patients with low back pain. Spine. 1998;23(22):2412–21. 9836355 10.1097/00007632-199811150-00011

[16] Michener L, Snyder A, Leggin B Responsiveness of the numeric pain rating scale in patients with shoulder pain and the effect of surgical status. J Sport Rehabil 2011;20(1):115–28. 21411827 10.1123/jsr.20.1.115

[17] Chaffee A, Yakuboff M, Tanabe T Responsiveness of the VAS and McGill pain questionnaire in measuring changes in musculoskeletal pain. J Sport Rehabil 2011;20(2):250–5. 21576715 10.1123/jsr.20.2.250

[18] Duncan P, Weiner D, Chandler J, Studenski S. Functional reach: A new clinical measure of balance. J Gerontol 1990;45(6):M192–7. 2229941 10.1093/geronj/45.6.m192

[19] Rikli R, Jones C Development and validation of a functional fitness test for community-residing older adults. J Aging Phys Act 1999;7(2):129–61.

[20] Sakulsriprasert P, Vachalathiti R, Vongsirinavarat M, Kantasorn J Cross-cultural adaptation of modified Oswestry low back pain disability questionnaire to Thai and its reliability. J Med Assoc Thai 2006;89(10):1694–701. 17128846

[21] Fairbanks J, Couper J, Davies J, O’Brien J The Oswestry low back pain disability questionnaire. Physiotherapy 1980;66:271–3. 6450426

[22] Fritz J, Irrgang J A comparison of a modified Oswestry low back pain disability questionnaire and the Quebec back pain disability scale. Phys Ther 2001;81:776–88. 11175676 10.1093/ptj/81.2.776

[23] Foster N, Anema J, Cherkin D, Chou R, Cohen S, Gross D, Prevention and treatment of low back pain: Evidence, challenges, and promising directions. The Lancet 2018;391(10137):2368–83. 10.1016/S0140-6736(18)30489-6 29573872

[24] Beaton D, Hogg-Johnson S, Bombardier C Evaluating changes in health status: Reliability and responsiveness of five generic health status measures in workers with musculoskeletal disorders. J Clin Epidemiol 1997;50:79–93. 9048693 10.1016/s0895-4356(96)00296-x

[25] Stratford P, Binkley J, Riddle D Health status measures: Strategies and analytic methods for assessing change scores. Phys Ther 1996;76:1109–23. 8863764 10.1093/ptj/76.10.1109

[26] Breivik E, Björnsson G, Skovlund E A comparison of pain rating scales by sampling from clinical trial data. Clin J Pain 2000;16:22–8. 10741815 10.1097/00002508-200003000-00005

[27] Bohannon RW, Bubela DJ, Magasi SR, Wang Y-C, Gershon RC Sit-to-stand test: Performance and determinants across the age-span. Isokinet Exerc Sci 2010;18(4):235–40. 25598584 10.3233/IES-2010-0389 PMC4293702

[28] Weiner DK, Bongiorni DR, Studenski SA, Duncan PW, Kochersberger GG Does functional reach improve with rehabilitation? Arch Phys Med Rehabil 1993;74(8):796–800. 8347063 10.1016/0003-9993(93)90003-s

